# Silent Danger: Retroperitoneal Air as a Radiological Red Flag

**DOI:** 10.1055/a-2667-6955

**Published:** 2025-08-18

**Authors:** Jakob Posch, Lucia Maria Winkler, Christos Vlachos, Michael H Fuchsjäger, Emina Talakić

**Affiliations:** 1 Department of Radiology 31475 Medical University of GrazGrazStyriaAustria; 2 Department of Surgery 31475 Medical University of GrazGrazStyriaAustria

## Introduction

Free retroperitoneal air due to perforation of the descending colon is a rare but life-threatening clinical entity. While colonic perforations are commonly associated with diverticulitis, malignancy, or fecal impaction, their isolated retroperitoneal manifestation is distinctly uncommon and sparsely documented in the literature. The descending colon, due to its secondary retroperitoneal anatomical location, may perforate without causing classical peritoneal signs, often delaying clinical recognition and surgical intervention 1 . Affected patients frequently present with nonspecific symptoms such as mild abdominal discomfort, subcutaneous emphysema, or even mediastinal air – findings that may only be revealed through advanced imaging 2 . Due to the subtle clinical presentation, diagnosis is challenging and often relies on repeat cross-sectional imaging. Computed tomography (CT) remains the gold standard modality, allowing for detection of retroperitoneal free air and associated findings like localized fat stranding or bowel wall thickening. Although the exact incidence of retroperitoneal colon perforation is unknown, it is considered exceptionally rare, with only isolated case reports described to date 3 . The overall mortality for colonic perforation is reported to be up to 17%, and delayed diagnosis in retroperitoneal cases may further worsen outcomes. This case highlights the importance of considering gastrointestinal perforation in elderly patients with atypical presentation and emphasizes the critical diagnostic role of CT, especially if clinical suspicion persists.

## Case Presentation

A 90-year-old female patient was referred to the gynecology department with suspected vaginal bleeding. Upon clinical examination, the source of bleeding was identified as rectal. Vaginal palpation revealed a firm resistance in the dorsal region, initially raising suspicion of a pelvic mass. The abdomen was soft and non-tender, without any signs suggestive of peritonitis. Laboratory findings demonstrated elevated inflammatory markers, prompting further evaluation. The patient was subsequently transferred to the surgical department.

**Fig. 1:**
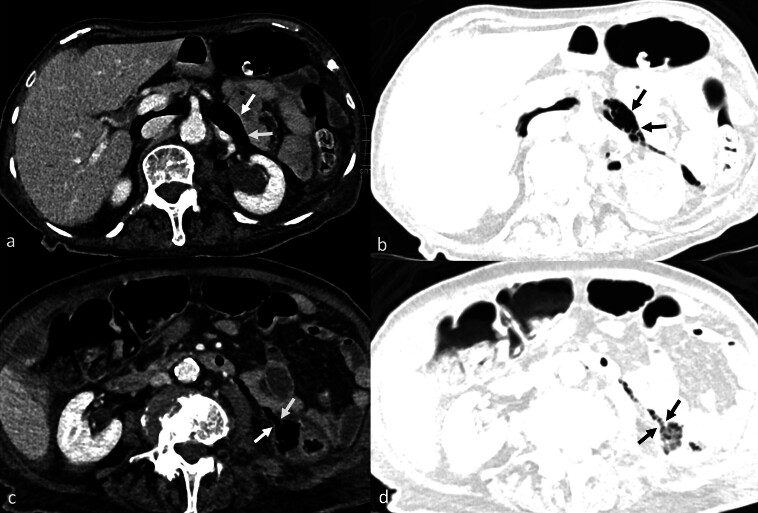
Axial CT images of the abdomen. **a, b** : Soft tissue and lung window images demonstrate free retroperitoneal air predominantly along the left colon (arrow). **c, d** : The suspected site of perforation (arrow) is visible in the distal descending colon.

Contrast-enhanced CT of the abdomen was performed. While no evidence of bowel obstruction or pneumoperitoneum was detected, imaging revealed pronounced fecal impaction in the descending colon ( Fig. 1 ).

**Fig. 2:**
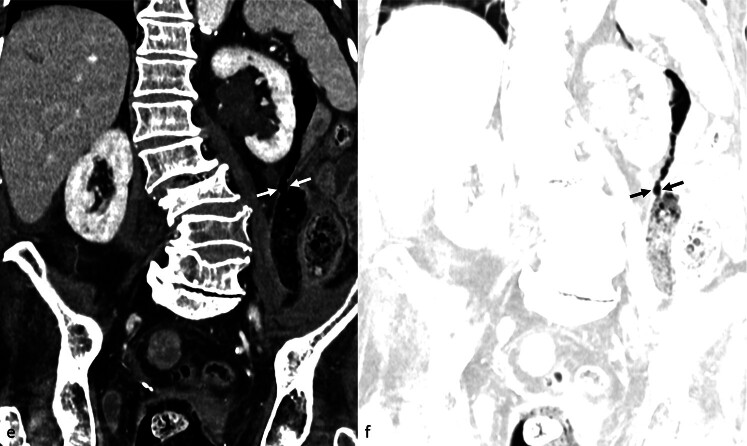
Coronal CT images of the abdomen. The site of perforation (arrows) in the descending colon is clearly visualized, with surrounding retroperitoneal gas extending along the left paracolic gutter.

Despite conservative management with intravenous antibiotics and stool softeners, inflammatory parameters remained elevated, and the patient developed progressive abdominal discomfort. On the fourth day of hospitalization, follow-up CT was conducted due to worsening clinical status. This examination demonstrated newly developed retroperitoneal free air along the left colon, highly suggestive of a contained colonic perforation ( Fig. 2 ).

An emergency laparotomy was performed, confirming multiple small perforations in the distal descending colon, associated with surrounding phlegmonous inflammation and impacted fecalomas. A left hemicolectomy with sigmoid resection and end-transverse colostomy was carried out. Histopathological analysis revealed extensive transmural inflammation without evidence of malignancy.

**Fig. 3:**
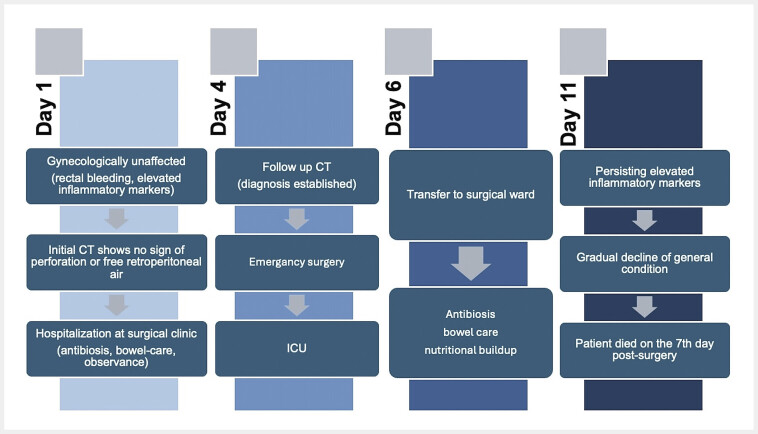
Clinical timeline of the patient’s course over 11 days, from initial presentation to postoperative death.

The patient was admitted to the intensive care unit (ICU) for postoperative care. Despite broad-spectrum antimicrobial therapy, intensive supportive measures, and nutritional management, her condition continued to deteriorate. She succumbed to septic complications on postoperative day 7. A timeline summarizing the patient’s clinical course is presented in Fig. 3 .

## Discussion

Free retroperitoneal air resulting from perforation of the descending colon is an uncommon finding. While its iatrogenic manifestation is observed more frequently – in case of ERCP 29% of patients show small amounts of free retroperitoneal air – manifestation resulting from traumatic, inflammatory or malignancy-related causes are rarely documented 4 .

Concerning diagnostics, plain radiography is often the first-line approach in day-to-day clinical settings but shows severely limited efficacy in identifying free retroperitoneal air. While sonography is commonly used to assess abdominal pathologies – especially in emergency settings – it is also subject to limitations to definitively diagnose free retroperitoneal air and – more often than not – follow-up CT is required. Therefore, CT is considered the gold standard for assessing free peritoneal/retroperitoneal air. Moreover, CT can reveal additional related findings, such as duodenal wall thickening or mesenteric fat stranding, which can assist in diagnosing gastrointestinal perforations 5 .

## Conclusion

Retroperitoneal perforation of the descending colon is a rare but potentially life-threatening condition. Clinically as well as radiologically its presentation is often subtle, which can delay diagnosis. This case highlights the importance of maintaining a high level of clinical suspicion in elderly patients presenting with vague abdominal or rectal symptoms. Repeat CT imaging plays a critical role for timely detection, and early surgical intervention remains essential to improving patient outcomes.
